# Can i have a second child? dilemmas of mothers of children with pervasive developmental disorder: a qualitative study

**DOI:** 10.1186/1471-2393-10-69

**Published:** 2010-10-26

**Authors:** Miyako Kimura, Yoshihiko Yamazaki, Mieko Mochizuki, Tomoko Omiya

**Affiliations:** 1 Department of Health Sociology, Graduate School of Health Sciences and Nursing, The University of Tokyo, 7-3-1 Hongo, Bunkyo-ku, Tokyo 113-0033, Japan

## Abstract

**Background:**

Pervasive developmental disorder (PDD) has an uncertain etiology, no method of treatment, and results in communication deficiencies and other behavioral problems. As the reported recurrence risk is 5%-10% and there are no methods of either prevention or prenatal testing, mothers of PDD children may face unique challenges when contemplating second pregnancies. The purpose of this study was to explore the mothers' lived experiences of second child-related decision-making after the birth of a child with PDD.

**Methods:**

The participants for this study were restricted to mothers living within the greater Tokyo metropolitan area who had given birth to a first child with PDD within the past 18 years. The ten participants were encouraged to describe their experiences of second-child related decision-making after the birth of a child with PDD on the basis of semi-structured interviews. Data analysis was performed by using Interpretive Phenomenological Analysis (IPA), which is concerned with understanding what the participant thinks or believes about the topic under discussion.

**Results:**

We identified two superordinate themes. The first was balancing hopes and fears, in which hope was the potential joy to be gained by the birth of a new child without PDD and fears were characterized as uncertainty of PDD and perception of recurrence risk, burden on later-born children, and negative effects on a child with PDD.

The second superordinate theme was assessing the manageability of the situation, which was affected by factors as diverse as severity of PDD, relationship between mother and father, and social support and acceptance for PDD. Our 10 participants suffered from extreme psychological conflict, and lack of social support and acceptance for PDD created numerous practical difficulties in having second children.

**Conclusions:**

Our participants faced various difficulties when considering second pregnancies after the birth of children with PDD in the Japanese society. As lack of social support and acceptance for PDD also played a large role in second child-related decision-making, creating a social environment that more fully accepts those disabled and providing flexible support systems for families of children with PDD are crucial.

## Background

Pervasive developmental disorder (PDD) refers to a group of disorders, namely, autistic disorder, Asperger's disorder, pervasive developmental disorder not otherwise specified, Rett's disorder, and childhood disintegrative disorder [1], although the first three are also classified as autistic spectrum disorders (ASD) [2]. These disorders are characterized by qualitative abnormalities in reciprocal social interactions and patterns of communication and by restricted, stereotyped, repetitive repertoire of interests and activities [3]. Current best estimates of prevalence for PDD are around 60 to 70/10,000, and PDD could be regarded as one of the most frequent childhood neurodevelopmental disorders [4]. Genetic factors are considered about 90% responsible, but specific causes can be identified in only 5%-10% of cases [5], and the etiology of PDD remains largely unknown.

Many studies have shown how these disabilities cause significant stress for families [6,7], exacerbated by factors such as time required for a firm diagnosis, difficulty in managing behavioral problems, intractability, and lack of social understanding [8]. Indeed, parents of children with autism/PDD have been shown to endure greater stress than parents of either children without PDD or those with other disabilities [9,10], and siblings are more likely to display behavioral and emotional problems as well [11,12]. In addition, recurrence risk for PDD/ASD has been estimated at 5%-10% [13-15]. As a result, parents of children with PDD tend to be overly worried about risks for later-born children [14], often overestimating actual recurrence risks [13,16].

Furthermore, traditional gender roles remain, and this results in mothers largely being responsible for child-rearing [17]; thus, mothers of children with autism/PDD show higher levels of stress and negative effects than fathers [18,19] as well as higher risks for depression, isolation, exhaustion, and stress than mothers of children with other disorders [9,20]. However, although prenatal testing-related decision-making has been relatively well studied for Down's syndrome and other congenital disorders, decision-making about subsequent pregnancies among mothers of children with PDD has been virtually ignored as a subject of study. The reasons for this may include the fact that the etiology of these diseases remains largely unknown, making prenatal diagnosis impossible, and that the long period of time required for a firm diagnosis leads parents to plan second pregnancies before even becoming aware of their first child's disability. However, it is precisely the nature of these difficulties that makes it crucial to understand the psychological burden and other factors experienced by this group when considering second pregnancies.

In Japan, reported ASD/PDD has increased rapidly [21], and a recent study in Nagoya showed that the prevalence of PDD in the general population was estimated at 2.1% and the sibling incidence of PDD was 10% [15]. That is, the estimated prevalence of PDD was 210 per 10,000 population in Japan; thus, the prevalence of PDD is greater than the above averages of prevalence (60-70/10,000) [4]. Additionally, children with PDD and their families may face a harsher social environment in Japan than in Western countries. For example, in the United States of America, the Developmental Disabilities Services and Facilities Construction Amendments of 1970 and Developmental Disabilities Assistance and Bill of Rights Act of 1975 took effect in the 1970s, and the Americans with Disabilities Act of 1990 banned discrimination against disability [22]. On the contrary, in Japan, there was no system to aid those with developmental disorders prior to the enactment of the Law to Support Persons with Developmental Disabilities in 2005 [23], and there is still no law that bans discrimination against disability. In addition, owing to a lack of specialists and opportunities for support, Japanese parents are left to cope with anxieties, stress, and exhaustion surpassing that of other disabilities [24]. This is the situation despite the availability of social support, which plays an important role in the coping mechanisms of the parents of autistic children [25]. Furthermore, mothers of children with Down's syndrome in Japan tend to be forced to take an amniotic test by other mothers in the same situation [26] and pressured to abort their fetuses by family members and doctors [27]. These cultural aspects may be important to elucidate decision-making surrounding subsequent pregnancy of mothers of children with PDD and the experiences they undergo. The purpose of this study was to explore the Japanese mothers' lived experiences of second child-related decision-making after the birth of a child with PDD.

## Methods

### Design

We considered a qualitative phenomenological approach to the experiences of this population the most appropriate for the current study given the lack of existing research and the sensitive nature of the issues involved. According to Phelps et al. [28], individuals with autism have unique characteristics, interests, and deficits, and their family systems are distinctive. Therefore, to grasp a richer understanding of the demands, needs, and experiences of caregivers who have children with autism, they used a qualitative phenomenological research design [28]. Similar to this, given the diversity of perceptions regarding PDD, we wanted to ensure that our methodology was capable of encompassing the significant variance among participants as well as the commonalities that we discovered. Interpretive Phenomenological Analysis (IPA) has been used to investigate the difficulties of reproductive decisions that individuals face and the ethical issues that society faces [29,30] and to explore the perceptions of young people with autism [31]. In addition, IPA is suited to researching in "unexplored territory," where a theoretical pretext may be lacking [32], and concerned with understanding what the participant thinks or believes about the topic under discussion and interested in exploring the nature of the gap that can exist between a situation/state and the individual's perception [29]. Thus, IPA seemed to be the most appropriate method for this study.

The population of potential participants for this study was restricted to mothers living within the greater Tokyo metropolitan area who had given birth to a first child with PDD within the past 18 years. We used purposeful sampling methods to recruit study participants through parents' groups and day service centers for developmentally disabled children. We were determined to recruit participants with first children younger than 18 years for several reasons (although we endeavored to recruit as many participants with elementary school age children as possible). First, childcare staff stated in preliminary interviews that inviting mothers of young children with PDD to participate might be ethically questionable as these mothers might not have had time to accept their children's disorder and are often themselves still emotionally vulnerable. Second, parents are often unaware of their children's disorders before they matriculate to elementary school. Third, mothers of children under the age of 18 will be knowledgeable about changes in offered social support systems over time. Finally, the timing of decision-making about second children has not been studied in this population, and we did not want to artificially eliminate a potentially useful segment from our study by setting too stringent a time limit since the birth of the first child. In Japan, the ideal number of children is overwhelmingly considered to be two [33], which is why we decided to focus on decision-making about second pregnancies. Those who expressed an interest in participation contacted the first author to learn more about the study and arrange a time and place for interviews. We then used snowball sampling to recruit more participants through word of mouth from this core group. As IPA focuses on analyzing language used by a small and homogenous sample [34], we decided on a maximum sample size of 10 participants.

### Data collection

Data collection was performed in one-on-one semi-structured interviews with the first author using a set of questionnaire and interview guide (additional file 1). The interview length ranged from 70 to 150 minutes, with a mean of 100 minutes. Interviews were performed from November 2007 to June 2008. Interviews consisted largely of open-ended questions, focusing primarily on experiences from discovering the disability of the first child to decision-making about the second child. The interviewer responded flexibly with follow-up questions, but examples of initial questions include "Please tell me about your decision-making as to whether or not to have a second child" and "How do you feel about that decision-making from your current perspective?"

This study was approved by the Institutional Review Board of the University. Prior to the interviews, the author explained the purpose of the study in depth in order to obtain informed consent, which included permission to record interviews.

### Analysis

Throughout the interview, the interviewer took note of each interviewee's stressed comments, frequently used words and expressions, pauses, tones, laughter, repetition, and degree of fluency, all of which were used to analyze the recorded data. These indicators were needed to grasp how the transcript illustrated the context and meaning and how metaphors could link descriptive and conceptual comments [34]. For example, when one participant frequently used the phrase "sibling of disabled" with tears or a smile and her subsequent comments contained very positive or negative expressions, we considered the phrase to be a metaphor for "fear" and "hope," two contradictory feelings. The interviewer then focused on how the next participant used similar expressions with similar or different meanings. This process thus gave clues for further developing the content of the interviews, helped indentify common experiences and common perspectives and differentials among participants, and led to the emergence of themes. In detail, we followed guidelines for IPA [34], including creating and analyzing verbatim transcripts from interview recordings. The first author 1) read the transcript multiple times, made initial comments on the text, and transformed these into themes capturing the "essential quality" of the text, 2) analyzed the relationships among these themes until they formed a consistent whole, resulting in an ordered list of themes that allowed us to identify the superordinate and subordinate themes for each subject, 3) after doing this for all interviews, identified shared superordinate themes for the group as a whole, confirmed by analyzing relevance to subordinate themes, original comments, and actual language used in the interviews, and 4) added descriptive commentary. The second, third, and last authors independently confirmed the appropriateness of coding, themes, and analyses at each stage, and provided alternative ideas about the first author's work. To offer a more objective commentary, our work was presented in meetings with researchers familiar with health sociology, disabilities, nursing, and qualitative research, and we used their feedback to revise the final theme, commentary, and analyses. For example, in the meeting, these researchers pointed out that the relationships between the participants' comments and their backgrounds, family relationships, usefulness of social support, and current situations should be fully reflected in our interpretations, and the hidden meaning of the words had to be more deeply grasped. To respond to these, we drew a map for each participant, which included the participant's background, family relationships, availability of social support, severity of disability related to each other, and how such relationships were reflected in their comments and decision-making. These maps were helpful to gain insight into the interaction between various social contexts and the participants' decision-making. When we faced the difficulty of interpreting the participants' comments or needed additional information, we contacted each participant by using e-mail or telephone, and obtained further explanations or information. After all analyses were completed, the authors returned to the mothers who participated in the study in order to confirm our own findings.

There were no complaints about our interpretations, and we obtained very positive comments from the participants. The feedback from these participants confirmed the validity of our descriptions of participants' experiences.

## Results

### Findings

The participant age range was 37-47 (mean 42) years, whereas the first child age range was 7-15 (mean 10) years. At the interview, four out of the 10 participants had second children, two of whom also had PDD. Of the remaining six, three participants made active choices not to have second children, one is still undecided, and the remaining two desired second children but were unable to become pregnant. Nine out of 10 mothers were still married, while one was divorced following the birth of her first child. Six participants were full-time homemakers, while four worked part-time. None had full-time jobs. Participant characteristics are shown in Table 1.

**Table 1:** Participant characteristics

Participant	Sex of child with PDD and sibling		Marital status	Occupational status
A	■	□	Married	Part-time worker
B	■	■	Married	Full-time homemaker
C	■		Married	Part-time worker
D	■	■	Married	Full-time homemaker
E	■		Married	Full-time homemaker
F	■		Married	Full-time homemaker
G	■	○	Married	Full-time homemaker
H	●		Married	Full-time homemaker
I	●		Married	Part-time worker
J	■		Divorced	Part-time worker

■male with PDD □male without PDD ●female with PDD ○female without PDD

Two superordinate themes emerged from the descriptions of mothers of children with PDD when considering second pregnancies. The first was balancing hopes and fears, which included four subordinate themes: uncertainty of PDD and perception of recurrence risk, burden on later-born children, negative effects on a child with PDD, and the potential joy to be gained by the birth of a new child without PDD.

In other words, the mothers were caught between the hope that a new child without PDD could help improve their current status and fears about the multitude ways in which a new child could exacerbate the current situation. The second was assessing the manageability of the situation, which included three subordinate themes: severity of PDD, relationship between mother and father, and social support and acceptance for PDD. These influenced the balancing of hopes and fears, and decision-making of the participants through determining whether or not the available environment was conducive to a second child, and whether or not the mother herself could manage given the constraints imposed on her (see additional file 2).

### Balancing Hopes and Fears

This superordinate theme epitomizes the psychological conflict mothers of children with PDD face when contemplating a second child. All participants in the study originally hoped for multiple children, with two children as "normal," "usual," and "expected." However, once they learned of their first child's disabilities and obtained more information from other mothers at education/care centers, the decision to have second children became more complex.

### Uncertainty of PDD and perception of recurrence risk

Most mothers visited health professionals multiple times around the child's first birthday because of suspicions that something was wrong, yet received little help, often being told that "everything is fine," "it all depends on how you raise the child," and "we cannot tell anything yet." For this reason, most participants described the uncertain nature of the disability as one of the most challenging aspects. Most participants also began actively considering second children at this stage, but lack of a firm diagnosis about their first children and the excessive amount of work involved in caring for them precluded any easy decision to have second children. Thus, participants began to attend education/care centers, some of whom did so before receiving a definitive diagnosis about their children. For these mothers, witnessing the other children with PDD was sufficient to convince them that their own children were developmentally delayed as well, and that they themselves were also the mothers of a "disabled child," a realization that came as a significant shock. In addition, most participants became aware of the risk for recurrence.

*Participant (P): Once I started bringing my child to education/care centers after discovering he was autistic, so many mothers there only had one child...There were also lots of mothers there whose first children were autistic, and their second children had autism or other developmental disorders as well. So, I began to think of what would happen if my second child were disabled as well*.

*Interviewer (I): You didn't know the recurrence risks*...

P: Not at all...I saw some children with developmental disorders, but I never thought it could happen to my own child...so I was scared...I couldn't imagine our future...yeah...I was so scared...(Mother E; one child only)

This description illustrates typical ways in which mothers learned of the risk of recurrence and how they came to consider a potential second pregnancy. Similar to Mother E, most participants were overwhelmed with fundamental uncertainties surrounding PDD. What were the chances of a second child developing it as well? What did that mean for the future? Although most of the participants had at least some knowledge of Down's syndrome, they had almost no level of familiarity whatsoever with autism or other forms of PDD. Indeed, until their own children began displaying problematic behavior, these mothers lived in a world completely removed from PDD and its attendant worries. The next participant, Mother I immediately decided against a second child after hearing rumors about recurrence risk.

I: When did you first start considering a second child?

*P: During my first pregnancy*.

I: What were your thoughts at the time?

*P: At that time, I was 35 years old...so I wanted to have a second child as soon as possible. But after the birth, I had to take care of my first child and I couldn't have a second baby soon. And...nearly three years later, my child was diagnosed as PDD...When I realized my child had a disability, and heard from another mother in the same situation that a second child would also have a high probability of disability, I decided not to get pregnant again. The probability of the second child also being disabled is, what, 50-60%? So, I have no regrets now about deciding not to have a second child*.

I: What are the reasons for that decision on your part?

P: The reason for my decision is discrimination against the disabled. Whatever people say, the disabled are discriminated against. Japan as a society isn't an easy one for the disabled to live in. (Mother I; one child only)

Mother I experienced no indecision about giving up the idea of a second child after hearing about the risk of recurrence. This participant's own discrimination closely mirrored that of society at large, which she was criticizing. However, when asked whether or not she considered consulting a specialist about the risk of recurrence, mother I answered "I didn't bother, because I knew I wouldn't get the only answer I wanted anyway (100% chance of a child without PDD)." Similarly, the majority of participants did not consult a specialist about the possibility of the risk of recurrence, and so came to the conclusion that it was "high" after speaking with other mothers in similar situations. Further, participants were not motivated to look for answers about the risk of recurrence in books or Internet resources because of the lack of material about the participants written for a lay audience in Japanese. Instead, most participants responded that "Asking other mothers (of children with PDD) was both faster and more reliable."

Mother A was the only mother who actually consulted a pediatrician about the risk of recurrence, although she did not obtain scientific information.

*P: I had no significant relationships with mothers of other disabled children when contemplating my second pregnancy, but I noticed that many mothers in similar situations did not have second children*.

I: Yeah...uh...Did you talk about your plans for second pregnancy with mothers in similar situations...?

*P: No, No, I couldn't...but...um...I felt something strange...something wrong...so I consulted my pediatrician about the risk of the second child being disabled as well*.

I: Did you?...well...What did your pediatrician say?

*P: My pediatrician said there was almost no chance that my second child also would be disabled and that I should hurry up and give my first child a sibling (laughter)*.

I: Really? Hum...uh...How did you feel?

*P: I...I was relieved (laughter). I just believed him and thought the probability was very low. Of course, I did hear about the "high" recurrence risk from another mother of a PDD child after my second child was born (laughter)*.

I: Oh...How can I say this (laughter)...umm...What do you think about your pediatrician?

P: Maybe he didn't know about the recurrence risk (laughter), or maybe he lied to encourage me. Either way, my second child is healthy, so I'm glad that I got pregnant again without knowing the truth [about the recurrence risk]. (Mother A; second child without PDD)

Mother A expressed gratitude towards the pediatrician who downplayed the very possibility of the risk of recurrence probably because if he had not, fear would have prevented her from having a second child, and she would have deprived herself of the experience of raising a child without PDD.

### Burden on later-born children

The participants also began hearing about negative effects on siblings without PDD from mothers in the same situation. Instead of an equal relationship in which siblings could be of assistance to each other, what now awaited younger siblings was the role of a "sibling of a child with PDD" and the unavoidable demands that are involved. Participants were nearly universal in expressing extreme hesitation in bringing this about.

*I: Please tell me about your decision-making as to whether or not to have a second child*.

*P:Ah...it's too complicated..I wanted to have a second child, of course, but I was afraid of burdens of later-born children*.

I: Burdens? What do you mean?

P: I felt that having a sibling with PDD would be unfair to a younger brother or sister without PDD. They would be forced to provide care, and I heard about bullying as well (sobbing).... (Mother A; second child without PDD)

Mother A's voice broke down as soon as she began speaking of her PDD child's "siblings." She gave birth to a normal second child, but continues to worry about the burden placed on this younger "sibling of disabled." Her main worries are that the second child will be bullied and be forced to provide care for the older sibling, sentiments shared by Mother F.

*P: When I noticed my son's disability, I almost gave up the thought of having a second child*.

I: Why did you think so?

*P: I have a friend whose older sister is intellectually disabled. I know how she has struggled in her own situation...it's too difficult*.

I: Did she complain about her circumstances?

*P: Not really...but...but I know that she has been patient. Please think about it*.

Parents and others always tell siblings of disabled children that everything will be alright, but in reality they know that they will end up having to care for their disabled brothers and sisters. It may be an obstacle to the sibling's own marriage, since the parents of the prospective spouse may object. I cannot help but think that it's very hard on the siblings. (Mother F; one child only)

These show how significant the issue of assumed burden on the later born children without PDD can be. In addition, many participants reported witnessing or hearing about "siblings with behavioral problems" and "siblings being deprived of attention" from other mothers in similar situations, and felt that having siblings with PDD represented a significant burden. Additionally, the burden takes the form of economic, psychological, and physical stress associated with caring for the disabled sibling after the parents' deaths, as well as the discrimination against family members of the disabled. As illustrated by worries about opposition to marriage with siblings of the disabled, Japanese society has difficulty accepting not only the disabled, but family members as well. Therefore, "If the second child has a disability, then things will be even harder on me, while if the second child is healthy, then having a PDD older sibling will be hard on them" (Mother I). Most participants shared similar feelings.

### Negative effects on a child with PDD

In addition to worrying about the burden placed on younger siblings, participants also contemplated the possible negative effects on their children with PDD brought about by the birth of younger siblings.

*P: I also thought that I should forgo having a second child so that I could look after my first child as well as possible. With a second child, my attention would be divided in half. That means I won't be able to give my older child as much care as before*.

*I: You mean...the existence of the second child might not be good for your first child*...

P: Yeah...I am worried about my first child's condition... (Mother A; second child without PDD)

This description is particularly representative of those participants who decided not to have a second pregnancy. As another participant said, "I want to give one child 100%" (Mother I). Indeed, several participants reported "...personally witnessing deterioration of the child with PDD after the birth of a younger sibling" (Mother D), a pattern making it even more difficult for mothers to have second children because of the possible negative effects on their own firstborn children with PDD. Here we see participants struggling with internal ideals of motherhood, which are complicated by the fact of their first child's PDD. Most participants reported feelings similar to "I'm so sorry for giving birth to a child like this," experiences which are likely tied to hesitation and guilt surrounding the decision to rear a second child. Moreover, one participant hypothesized about the direct negative effects on her child with PDD that a second child could bring.

*P: If my second child had PDD, it may have a negative impact on my son (first-born child)*.

I: What kind of negative impact?

*P: From pre-school on, my child has uttered self-denigrating statements, such as "Why did you have me?" "You would be better off without me," or "I want to erase myself." So, if my second child also had PDD, but of a different, opposite kind where they blamed everything on other people, then I think my first child might even commit suicide*.

*I: Oh...you imagined such*...

P: This could be happen. There are various types of children with PDD, and their behavioral problems change all the time. (Mother F; one child only)

Mother F is aware that although some children with PDD are extremely critical of and even harm themselves, the opposite type, with aggression directed outward, occurs as well. She is therefore fearful that her current child with PDD might be driven to extremes if her second child developed this second type of PDD. All participants were aware of the fact that expression of underlying PDD varies significantly according to the individual, and that even individual symptoms vary over time: "He just stopped talking one day," or "He was such a gentle child, then suddenly he began hurting others." These realizations lead to fears about uncertainty of how PDD might be expressed in a second child with PDD, and the possibility that differing forms of PDD within the same family might exacerbate the situation.

### The potential joy to be gained by the birth of a new child without PDD

In contrast to the fears described above about giving birth to a second child, nearly all participants had a desire for children without PDD. This desire was different from that experienced before learning of their first child's disability, and could be better described as a fervent hope for an opportunity to improve their current situation, alter their lives, and reclaim lost dreams.

I: You have a second child...When you think about your second pregnancy?

*P: When I realized that autism is never cured, I couldn't accept such a life of dealing one-on-one with a child who never spoke... Then I thought that having a second child give me only opportunity to escape such life*.

I: You mean, having a second child may improve your situation?

P: Yes. I really want to raise a healthy child, and experience normal life. My husbands' family members, his mother and brother, always blamed me for my child's behavior. They didn't understand the nature of PDD, and...and...Can you believe this? They had not accepted my son's disability for the past 12 years. (Mother B; second child with PDD)

In the case of Mother B, the difficulties posed by her child only strengthened her resolve to have another child. In addition, as the husbands' family members denied Mother B's ability to raise a child a long time ago, she struggled to escape from her situation. Similarly, in the case of Mother D, her husband's father, despite being a doctor himself, has yet to accept the nature of his 11-year old grandchild's condition, continually insisting that the mother's childrearing is at fault. With the husband's support limited to telling her to "ignore what [the grandfather] says," Mother D is extremely isolated within her husband's family. According to Mother B, "causes are not sure, disability is invisible at first sight, and diagnosis needs long time, so accepting PDD should be difficult for family members, especially for grandparents. They had wanted to treat my son as a healthy, normal, and ideal grandchild." These may result in blame against mothers, and increasing mothers' wishes to change the current circumstances by giving birth to a child without PDD.

*P: I never considered disabilities as something special, but uh...wanted to have a second child who would be normal (laughter)*.

I: Yeah...what do you think about your second pregnancy?

P: I was looking forward to reading children's books and doing other things for my child. But, my child won't listen to me read, and tears up the books. It's hard to communicate...I don't get to feel the typical joys of raising a child. So, I decided to gamble on a second child, in the hopes of being able to experience the fun and joys of raising a child without PDD. (Mother J; one child only)

Mother J considered reading books aloud integral to and metaphor of the joys of raising her own child. Her unexpected inability to do so only heightened her sense of loss for the "joys of raising a child without PDD." Klaus and Kennel [35] describe the process of accepting children with congenital disabilities as involving feelings of loss for "expected healthy children," and "normal children." PDD is not diagnosed at birth, so participants spared the shock and grief of learning of their child's disorder until later. Instead, however, they were exposed to numerous situations in which the keen differences between their children with and without PDD were brought home to them. Many participants spoke of the pain involved in watching other parents and children participate meaningfully in school events without being able to share in the experience themselves because children with PDD are often uncontrollable and can disrupt the entire event: "At school plays or field-days, all the other parents were enjoying themselves and taking videos, but I just spent the time so nervous my child might create an incident that my stomach began to ache" (Mother H). These feelings of loss toward raising healthy children only heightened participants' desire for second children so that they might re-capture the opportunities they felt deprived of because of their first child's PDD.

### Assessing the manageability of the situation

This superordinate theme consisted of an assessment of their current situation on the part of the participants to determine if giving birth to and raising a second child was feasible. The feelings of helplessness experienced by participants engendered both hope and fear, sometimes influencing decision-making directly.

### Severity of PDD

In addition to the lack of communication that characterizes PDD, secondary behavioral disorders include self-injury, injuring others, sleep disorders, panic attacks, and hyperactivity. The presence of these often brought participants to the point of physical and psychological exhaustion.

*P: Making a second child would be impossible, both psychologically and in terms of time. I don't have time for myself, and I am so sleep-deprived that it's hard to stay awake...My child might do something if I fell asleep*.

I: So, you couldn't rest at all?

*P:Yeah...and his behaviour has changed all the time. Now, my son always blames himself, but when he was younger age, he was very aggressive. For example, he always hit me more than two hours, and once he broke my nose*.

I: Broke your nose?

*P: Yes, he did. I was so miserable.... Please imagine...I was an adult women, and the mother of my son. But he...just tiny infant, around three or four years old, made me cry and broke my nose*.

I: Oh...did you consult with specialists?

P: Yes, I consulted with a specialist of developmental disability, and she said, "You have to just be patient, as a stone." (Mother F; one child only)

Mother F has faced the difficulties that are not only her child's behavioral problems, but also uncertainty of PDD, which we already mentioned. Mother F was not able to imagine how her son's behavior would be changed and how she tackles him. That is, after she became the mother of child with PDD, her life was not able to be planned by herself. Therefore, in Mother F's case, the severity of PDD and uncertainty of PDD are intertwined and obstacle for planning to have a second child.

*I: You have wondered for a long time...but you still have time to make a decision about a second child*...

P: Yes, but it's almost too late (laughter). Certainly...yeah...I am not quite too old to give birth again, and I have not completely given up on the idea of a second child, but I'm already at the end of my rope, especially physically. My mother can only look after [my child with PDD] for an hour at a time, and I can't even go to the bathroom without worrying. One time I was busy with something else, and came back to find a cord wrapped around his neck. (Mother E; one child only)

Mother E's husband frequently looks after the child, and the grandmother helps out as well. However, the child is so hyperactive that even the grandmother can only watch the child for an hour at a time, leaving the mother completely lacking in time or resources. In cases such as these with high-level disorders, mothers' time and energy are both diminished to the point that participants despair of a second child as being "physically and psychologically impossible." This feeling of despair is closely linked to other subordinate themes, such as fears that if the second child has PDD as well the mother's exhaustion will double, while if the second child has not PDD he/she will bear a burden for their older sibling and the older sibling themselves may deteriorate. At the same time, the high level of severity only increases the desire to escape from the current situation in some way.

### Relationship between mother and father

All participants reported at least some change in relationships with their partners during and after discovering their child's disability. Although differing widely among participants, this relationship exerted an extremely large impact on decision-making.

*P: We can't go back to a romantic relationship, can't feel that way. Right now it truly takes everything I have to raise our child*.

I: When did your sexual relations end?

*P: Let's see...They probably truly ended when...our child was about three years old, I think*.

I: So... about four years ago?

P: Maybe...Yes, Yes. My husband and I were having problems as well when things truly hit bottom. I couldn't understand how he could be so aloof, and was frustrated that he didn't understand how I felt. (Mother F; one child only)

Mother F complained her husband's attitudes, which were cool and didn't express sympathy for her. However, although at one time she considered divorce, the relationship has now improved and she thinks of her husband as "a good source of advice," "a fellow soldier." This mother F' change was underpinned the increasing understanding for husband that even he shocked and wanted to cry, he couldn't do so with wife because of gender differences, and he must work outside with equanimity to feed his family. In other words, mother F became accepting that mother and father had different parenting roles and she should engage her son's care. However, the fact that the 37-year-old woman has had no sexual relationship with her husband for the past 4 years speaks to the great change wrought upon their relationship by their child with PDD. All participants reported at least some change in relationships with their partners during and after discovering their child's disability. Although differing widely among participants, this relationship exerted an extremely large impact on the decision-making. As typified by Mother F's statement that "His opinion is irrelevant... He isn't around on weekdays," more than half of the participants' husbands played a supportive role in child-rearing, but the mothers were left alone to care for their children with PDD on weekdays. For this reason, the mothers who bore the brunt of responsibility in making decisions about second children. The next case is similar to that of Mother F, except that they were unable to repair their relationship, with a permanent rift developing between them.

P: How about your ex-husband? Did he support you?

*I: Not at all...Rather, he shut himself into his own territory*...

P: Own territory?

*I: Yeah. He had his own world...He never tried to research our child's disability, and wasn't very interested. Then he started hitting our child when [the child] yelled, which was the start of our relationship becoming rocky. Although I might have paid too much attention to our child, too*.

*P: Oh...that must have been really difficult*...

I: Yeah...yeah...I decided to divorce him...It had nothing to do with my child's disability...I just no longer trusted him. (Mother J; one child only)

This illustrated that as the child grew older, the differences between the mother and father in terms of understanding PDD and deciding future plans widened, and these differences led to a breakdown in the marital relationship. Thus, despite the mother's strong desire for a second child, her marital relationship prevented her from fulfilling her wish.

In other cases as well, the husbands' words and actions were the source of significant stress for the participants. Mother B's husband simply rejected his son: "My husband stopped coming home, saying that our child was not his" (Mother B). However, these marital relationships, as well as the attitudes of the fathers, were subject to change over time, altering participants' perceptions of what was possible or impossible. A few years later, Mother B's husband eventually came to accept his child and became so actively involved that the participant was inspired to say that "It's OK if my next child is disabled too. These I thought, we will manage somehow" (Mother B). In direct opposition to Mother J, Mother B's case indicated the differences in the understanding of PDD between mother and father had decreased as time passed, and resulted in the decision to have a second pregnancy. Similarly, Mother A also describes the husband's cooperative attitude as a key element in alleviating her own burden and helping overcome inhibiting factors, such as the burden on the later-born child and negative effects on the first child. These illustrated how the husband's attitudes influence participants' assessment of their own ability to manage and the decision to have a second pregnancy.

### Social support and acceptance for PDD

Since available family supports were limited, the degree to which others and society in general offered social support and acceptance for children with PDD both directly and indirectly influenced the decision to have a second pregnancy.

I: Do you still want to have a second child?

P: Oh, it's too late (laughter)...I wanted someplace to take care of him temporarily, but not many places will look after disabled children. I was told that they have a hard time looking after such kids. There was only on public facility for looking after disabled children near me, and they had no openings. The government doesn't distribute that kind of information, either. It would be unthinkable to have another child without someone to look after my first child. I could have had another child with support. (Mother H; one child only)

Mother H was worried about both the burden of the later-born child and negative effects on the first child, but not enough to give up on the idea of a second child. Instead, it was the severity of PDD and lack of social support for children with PDD that directly prevented the decision to have a second child.

*P: He [first born child] loves babies*.

I: Really? Does he like to take care of other kids?

*P: Yes, he does...and...and my boy [with PDD] said that he wanted siblings. So, I said, who would look after you when I was in the hospital giving birth? What would we do if I had to stay in the hospital for a long time? There is no place willing to look after a child like that, and no one I can feel good about entrusting him to*.

P: Oh...that was a logical explanation (laughter). Did he understand?

I: Yes, for a while, he was just thinking (laughter). That time, he was a kindergarten boy...so he couldn't say more. (Mother F; one child only)

Mother F envisioned potential trouble if her second birth required a longer than anticipated hospital stay. She assumed that appropriate aid would be unavailable in her area, and lacked the energy to investigate the matter herself. She ultimately elected not to have a second pregnancy given worries about the burden on later-born child and negative effects on her child with PDD, which were compounded by severity of PDD, relationship between mother and father, and lack of social support and acceptance for PDD. Mother D reports that "the authorities take no initiative in providing information at all," a sentiment echoed by every mother interviewed: "[Government offices] will only give us the absolute minimum of information possible" (Mother C); "I read the public notices from cover-to-cover, and there is nothing about disabled children, not even that the system itself had changed" (Mother E); "Several years passed without us receiving the financial aid we were entitled to" (Mother D). All participants complained about the lack of social support and even information about what little support was theoretically available, an experience that heightened feelings of lack of social acceptance: "We are just social baggage anyway" (Mother B). Similarly, many other participants did describe the hardships faced by the disabled in current Japanese society: "People can be cruel to these children" (Mother H); "My child was so hurt by the words of his special needs teacher in elementary school that he refused to go to school afterwards" (Mother E). Perceptions of lack of social acceptance toward the disabled that accompanied these experiences only served to intensify anxiety about recurrence risk or burdens of later-born children.

*P: I wanted to go to a hospital, but I couldn't*.

I: You needed to seek medical treatment or something?

*P: I mean...I need fertility treatments to get a second child, but I couldn't bring my hyperactive child with me to the hospital for the treatment. I would have to work to put him in daycare, but no one would hire me, having to take so many days off for my fertility treatments and care for my child. And, there are so few spots for disabled children at elementary schools, and there are no after-school programs he could join anyway*...

I: I understand that depends on regions; only children of working mothers may be eligible for daycare centers...ah...are there no exceptions?

P: No exceptions for my area...um...I think, having a second child may be impossible...may be an impossible dream... (Mother E; one child only)

In Mother E's case, planning a second pregnancy through fertility treatments required greater flexibility in daycare eligibility requirements and other social services. An unavailability of such services may contribute to a sense of hopelessness; indeed, Mother E remains undecided about having a second child and regards it as "an impossible dream." This shows that negative appraisals of one's own situation overwhelm the desires for and preclude any possibility of having a second child.

## Discussion

Japanese mothers of first children with PDD face dilemmas, which center around themes of judging whether or not their situation will improve or deteriorate with a second child by balancing hopes and fears, and assessing whether or not such a child is feasible at all. According to Woodgate et al. [36], the Canadian parents with autistic children experienced "society's lack for understanding," "missing a normal way of life," "feeling disconnected from the family," and "the unsupportive system." As all of these are also found in our participants' experiences, parents of autism/PDD in Western countries and Asian countries may face similar difficulties.

However, our study may add different types of difficulties based on second child-related decision-making after the birth of a child with PDD in a Japanese society.

In Japan, 99.8% of all births take place in a hospital or clinic [37], and the average period of admission for childbirth, including recovery, is 7.6 days [38]. Thus, childcare for older children is necessary for approximately 1 week when younger siblings are born. It is not realistically feasible for most fathers to take a week of vacation to perform this role, as evidenced by the fact that not a single husband in our study group took a significant amount of time off for their wife's childbirth. In Japan, the normal recourse is to depend on grandmothers for support, though for various reasons this option is more problematic for children with PDD. As the difficulties of caring for children with PDD include uncontrollable behavior, it is too hard for grandmothers. There are some public facilities to fulfill this role, but they are few in number and information is so poorly disseminated that most participants were unaware of their existence. Further, mothers of children with PDD are highly unlikely to be able to withstand the mental, physical, and economic demands of fertility treatments. Japan has relatively few public daycare centers, and they often require that both parents work; meanwhile, private equivalents and babysitters are beyond the financial reach of most, even when equipped to accept disabled children. These factors contribute to the acute need for a system to care for children with PDD, while their mothers are either visiting the doctor or are admitted for childbirth. Although social support systems have improved in the few years since enactment of the new law [23], available support still differs drastically by region, and participants consistently complained of inflexibility of existing social support.

The lack of social support leads to feelings of lack of social acceptance, which in turn intensifies psychological anxiety. It has been noted that "Perception of disability is affected by the value system of the group or society in which one is raised and lives" [27], and indeed Mother I displayed difficulty in accepting her child's PDD in society. Youda [39] describes how the disabled are considered an "unacceptable presence" in Japanese society, and these discriminatory attitudes are rooted so deeply in modern Japanese society that the parents of disabled children themselves are "both the participants of and perpetrators of discrimination" [39]. The same trends have been observed in mothers of Down's syndrome children [26], and our own research.

Furthermore, there are gender differences. Gray [17] reports that mothers and fathers have different ways of accepting autism and that traditional gender roles remain in effect, with mothers largely responsible for child-rearing and fathers for work. Also, Woodgate et al. [36] explain that parents felt disconnected from their spouses when both experienced different feelings. Our study clearly illustrates that the same can be said of contemporary Japanese couples. When the participants' husband attempted to share the burden, the participants' psychological burden were mitigated and the marital bond was strengthened, so "positive transformation" [40] can be seen. However, the fathers did not, but the mothers experienced a widening marital gap and were left to engage their children alone.

There are clear differences between our findings and previous research into decision-making about second pregnancies by mothers of children with Down's syndrome. According to Tsuji [26], Japanese mothers with Down's syndrome babies viewed second pregnancies as "an opportunity to reclaim their self-worth" or to "create siblings for the sake of their Down's babies." However, in our study second children represented a way to escape participants' current situation, in which they were locked into a one-on-one relationship with a child incapable of true communication, and not a single participant stated a desire to have a second child for the sake of their child with PDD. Instead they overwhelmingly mentioned fears about second children being forced to care for their elder siblings, with over one-half of the participants reporting hesitation about second children on account of these worries. The difference between these two groups likely lies in the difficulty associated with caring for children with PDD. Parents of autistic children experience more stress than parents of children with Down's syndrome [9], and indeed a constant theme in our interviews was participants not wishing to push the exhaustion they themselves felt onto their second children. Underlying such a fear lies the assumption that siblings will be forced to care for those with PDD once the parents die. Further, whereas mothers of Down's syndrome children began planning second pregnancies as early as when their first children began walking or talking [26], the mothers in our group all made decision-making about second children relatively late. In fact, mothers in our study gave birth to second children 5 years (2 participants), 6 years (1 participant), and 7 years (1 participant) after the birth of their first child, although one participant remained undecided after as long as 10 years. In addition, our participants tended to express fears much more than hopes, suggesting that balance is difficult to achieve and causes of fear outnumber those for hope.

We were able to gain insight about our participants' perceptions of the risk of recurrence, a factor which to date had not been subject to study in Japan. The tendency in our country is not to offer scientific data about recurrence risk. Only one participant in the current study stated a concrete perceived recurrence risk probability at 50%-60%, but we found that most participants perceived recurrence risk to be high enough to feel hesitant about a second pregnancy. Similarly, in a recent study in Tasmania, one quarter of subjects thought recurrence risk to be higher than 60%, constituting a huge gap with the 5%-10% generally considered to be the actual recurrence risk for PDD/ASD [13-15]. As this suggests, the lack of objective, scientific data only nurtures unwarranted psychological fear on the part of concerned parents. However, although our participants required information about the recurrence risk, they were fearful of receiving a definite answer from the specialist. These complicated feelings may be shared by only mothers in the same situation; therefore, the participants rely on any information through mothers' network.

Whereas husbands, other family, and doctors are sometimes known to use amniotic test results to strongly pressure mothers to abort their fetuses [27], none of our participants described any pressure to give up on second children because of the risk of recurrence. As PDD itself is not a widely known or understood condition and the primary caregivers in this study are the participants themselves, family dynamic gives mothers the right to make their own decisions about second children.

### Methodological considerations & Limitations

IPA is regarded as a useful tool to hear the voices of participants from across the sociocultural spectrum and challenges the traditional linear relationship between sample size and value of research [32]. However, as we relied on parents' groups and word of mouth to recruit participants using snowball sampling, our sample is necessarily limited to those mothers who avail themselves of these kinds of support networks. This leaves unanswered important questions about the experiences of mothers without such support networks. In addition, we used only ten participants, and these participants were largely self-selected and may have had issues regarding second children, which nonparticipants may not. Furthermore, our research does not reflect the experience of mothers living in other, more rural regions, many of whom typically share dwellings with their husbands' families.

On the other hand, our findings, which described the difficulties faced by mothers of children with PDD may be useful to medical, educational, policy makers, social service, and other related professions, and improving the support services available should benefit both mothers and their children.

### Implications

To alleviate the fear of the recurrence risk, family planning centers, still relatively rare in Japan, as well as telephone counseling may be made more readily accessible. Similarly, in order to relieve worries such as burden on younger siblings, more social acceptance and support for the disabled are needed in the society. Moreover, an increased number of available social support systems such as daycare centers without onerous eligibility requirements, after-school programs, and paternal childbirth leave may be desirable. In addition to greater flexibility, greater dissemination of relevant information and education about the disabled as well as chances to interact with them from a young age are required.

## Conclusion

In conclusion, our participants who are the Japanese mothers of first-born children with PDD faced various dilemmas when considering second pregnancies. They balanced hopes that a new child could help improve their current situation against fears about the multitude ways in which things could deteriorate even further. Also, our participants assessed both whether their current environment was conducive to a second child and if they themselves could manage given current constraints. Therefore, our participants suffered from extreme psychological conflict, and lack of social support and acceptance for PDD created numerous practical difficulties in having second children in Japanese society.

## Competing interests

The authors declare that they have no competing interests.

## Authors' contributions

MK carried out the interviews, analysed the data and drafted the manuscript. YY, MM, TO participated in the design of the study and independently confirmed the appropriateness of interpretation and analysis of the data at each stage, provided alternative ideas about the first author's work, and helped to draft the manuscript. All authors read and approved the final manuscript.

## Pre-publication history

The pre-publication history for this paper can be accessed here:

http://www.biomedcentral.com/1471-2393/10/69/prepub

## Supplementary Material

Additional file 1 **Questionnaire and interview guide**. Questionnaire for interviewees and interview guide for an interviewer in this study Click here for file

Additional file 2 **Superordinate themes and Subordinate themes**. Superordinate themes and Subordinate themes derived from the interview data Click here for file
