# Supplementary material for: Can i have a second child? dilemmas of mothers of children with pervasive developmental disorder: a qualitative study
Source: BMC Pregnancy Childbirth. 2010 Oct 26;10:69. doi: 10.1186/1471-2393-10-69 (PMC2987885; doi:10.1186/1471-2393-10-69)
Supplement: Additional file 1 — Questionnaire and interview guide. Questionnaire for interviewees and interview guide for an interviewer in this study [file 1471-2393-10-69-S1.DOC]

***Questionnaire for interviewees***

Please complete following questions as well as possible

Q1 Your age

（　　　　　　）

Q2 This section, please answer your child’s status (Circle one OR fill in detail)

Birth order　　age　　 　sex　　　 Does he/she have disability?　　　 Details of disability

First （　　　）　（male　female）　 （Yes　No） （　　　　　　　　　　　　　　　　　）

Second （　　　） 　（male　female）　 （Yes　No）　 （　　　　　　　　　　　　　　　　　）

Third　 （　　　）　 （male　female） 　 （Yes　No） （　　　　　　　　　　　　　　　　　）

Fourth （　　　） 　（male　female）　 （Yes　No）　 （　　　　　　　　　　　　　　　　　）

Other （　　　）　（male　female）　 （Yes　No） （　　　　　　　　　　　　　　　　　）

Q3　Your occupational status

1　Full time job　　　2　Part time job　　　　3　Self-employed　　　　4　house-maker

5　Student 6 Other　(　　　　　　　　　　　　　　　　)

Q4　Family member (living with you) （Circle）

１．Nobody　 ２．Spouse 　　 ３．Sibling 　 ４． Parent ５．Parent-in-law

６．Other （　　　　　　　　　　　　　　　　　　　）

Q5　Marital status (Circle one)

1. Single　　　　2. Married　　　　3.　Divorced　　　　4.　Widowed

Q6　Economic status (Circle one)

１．Excellent　 　２．Very good　　 　３．Good 　　　 ４.Fair ５．Poor

Q7　In general, would you say your health is (Circle one)

１．Excellent　 　２．Very good　　 　３．Good 　　　 ４.Fair ５．Poor

Q8 What is your education level? （Circle one）

１．Junior high graduate 2. High school graduate 　３．Technical college graduate

４．Junior college graduate　　　　５． College graduate ６．postgraduate school graduate

７．Other ( )

Thank you for your help!

***Interview guide for an interviewer***

1. What can you tell me about the timing of learning of your first child’s diagnosis?

--- How you felt at the time?

--- How about your partner?

--- How about other family member?

1. When did you first start considering a second child?

--- What were your thoughts at the time?

--- How about your partner?

--- How about other family member?

1. Were you conflicted about having a second child? If so, in what way?

--- How about your partner?

--- How about other family member?

1. Please tell me about your decision-making as to whether or not to have a second child?

--- How about your partner?

--- How about other family member?

1. How do you feel about that decision-making from your current perspective?
2. Do you have any problems now?
