# Supplementary material for: Can i have a second child? dilemmas of mothers of children with pervasive developmental disorder: a qualitative study
Source: BMC Pregnancy Childbirth. 2010 Oct 26;10:69. doi: 10.1186/1471-2393-10-69 (PMC2987885; doi:10.1186/1471-2393-10-69)
Supplement: Additional file 2 — Superordinate themes and Subordinate themes. Superordinate themes and Subordinate themes derived from the interview data [file 1471-2393-10-69-S2.DOCX]

**Superordinate themes and Subordinate themes**
